# Quantitative High-Resolution Genomic Analysis of Single Cancer Cells

**DOI:** 10.1371/journal.pone.0026362

**Published:** 2011-11-30

**Authors:** Juliane Hannemann, Sönke Meyer-Staeckling, Dirk Kemming, Iris Alpers, Simon A. Joosse, Heike Pospisil, Stefan Kurtz, Jennifer Görndt, Klaus Püschel, Sabine Riethdorf, Klaus Pantel, Burkhard Brandt

**Affiliations:** 1 Department of Tumor Biology, University Medical Center Hamburg-Eppendorf, Hamburg, Germany; 2 Center for Bioinformatics, University of Hamburg, Hamburg, Germany; 3 Bioinformatics, University of Applied Sciences Wildau, Wildau, Germany; 4 Department of Legal Medicine, University of Hamburg, Hamburg, Germany; Roswell Park Cancer Institute, United States of America

## Abstract

During cancer progression, specific genomic aberrations arise that can determine the scope of the disease and can be used as predictive or prognostic markers. The detection of specific gene amplifications or deletions in single blood-borne or disseminated tumour cells that may give rise to the development of metastases is of great clinical interest but technically challenging. In this study, we present a method for quantitative high-resolution genomic analysis of single cells. Cells were isolated under permanent microscopic control followed by high-fidelity whole genome amplification and subsequent analyses by fine tiling array-CGH and qPCR. The assay was applied to single breast cancer cells to analyze the chromosomal region centred by the therapeutical relevant *EGFR* gene. This method allows precise quantitative analysis of copy number variations in single cell diagnostics.

## Introduction

During cancer formation and progression, cell populations with distinct genetic aberrations arise that represent unique clinical entities harbouring specific therapeutic targets [1], [2]. A prominent example is the gene locus of the epidermal growth factor receptor (*EGFR*), which is a key player in tumor biology and an important target for individualized cancer therapy. The DNA copy numbers for this single locus significantly determine the phenotype of cancer cells and are indicators for patient's response to chemotherapy or radiotherapy [3]. Antibodies and small molecule inhibitors have been developed impairing EGFR tyrosine kinase activity in various tumor types [1]–[3].

Most tumors can be completely removed by surgery and are therefore unavailable for repeated sampling to monitor either treatment response or treatment-induced changes in genomic aberrations or disease progression, respectively. Recently, these crucial processes can be assessed by the detection of blood-borne cancer cells or disseminated tumor cells (DTCs) in bone marrow, as prominent homing organ and major site of overt metastases in cancer patients. The molecular analysis of these cells may reveal unique information to tailor therapies preventing metastatic progression [4], [5].

Therefore, we developed an approach for a reliable high-resolution quantitative genetic analysis of isolated single tumor cells on the example of the *EGFR* gene. After enrichment, cells were isolated by micromanipulation and subsequent linear whole genome amplification was performed. Evaluation of this procedure has been done by fine-tiling array-CGH and quantitative PCR in order to accurately determine amplitude and extension of the *EGFR* amplicon in single tumor cells.

## Results

### Micromanipulation and whole genome amplification of single cells

The human mammary adenocarcinoma cell line MDA-MB-468 was selected as suitable model for method evaluation, since it harbors an *EGFR* amplification and shows strong EGFR overexpression as well as displays a stemness/committed progenitor cell phenotype [6], [7]. However the level of amplification varies between cells. For whole genome amplification (WGA), non-fixed or slightly fixed cells were collected from the glass surface using a micromanipulator equipped with a capillary designed to our specific requirements. Cells were transferred in an aqueous surrounding onto a C_18_-silane-coated glass stick carrying a spot of dried complete cell lysis buffer (see online methods). The cell lysate on the glass stick was directly transferred into a reaction tube already prepared with the sample buffer for WGA to avoid any loss of genomic material. The procedure is based on multi-displacement amplification by the use of random hexamers and the proofreading DNA polymerase Phi29 to ensure linear amplification [8]. A single cell yielded in 2.04–2.96 µg PCR-amplifiable DNA (average: 2.42 µg, SD 0.26), which is superior to the results previously described [9].

Transfer efficiency of micromanipulation and fidelity of the WGA of one single cell including the *EGFR* locus of approximately 4.5 MB were validated with microsatellite PCR. Microsatellite loci have been adapted from Tidow et al. [10] and detailed information is provided as supplementary information (**Table S1, S2**). All microsatellite loci could be verified.

### Description of the EGFR amplicon in single cells by fine tiling array CGH

**Figure 1:**
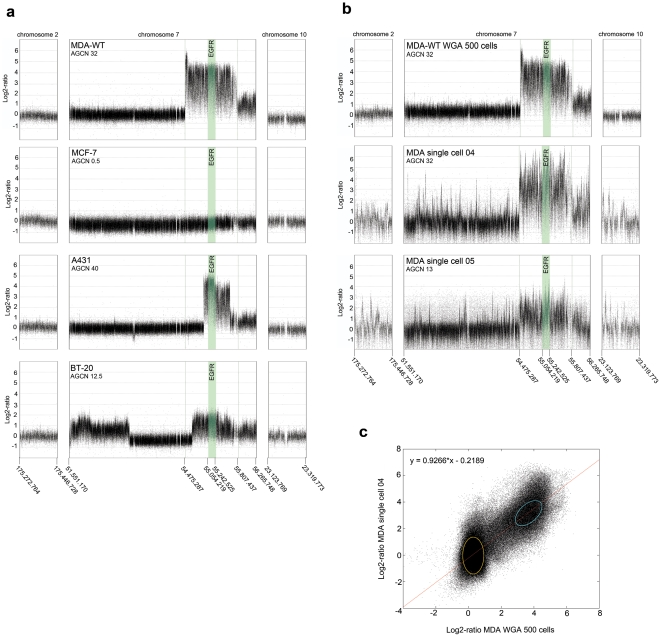
Fine-tiling array-CGH analyses. **a**: Array-CGH plots of genomic DNA of different breast carcinoma cell lines with different *EGFR* gene copy numbers. **b**: Array-CGH plots of WGA amplified DNA from 500 MDA-MB-468 or single MDA-MB-468 cells (purchased at ATCC), respectively. The region of the *EGFR* gene is depicted in green. **c**: Correlation plot of the signal intensities after array hybridization comparing the signals of the DNA from one single MDA-MB-468 cell to the signals obtained from the DNA isolated from 500 MDA-MB-468 cells. Spots depicted in yellow represent the signals outside the amplicon region, whereas spots indicated by the blue color represent the signals obtained by the amplicon region on chromosome 7.

To control for linear amplification of the DNA sequences by WGA and to investigate the structure of the *EGFR* amplicon, we analyzed the region harboring the *EGFR* gene by a high-resolution two-color fine-tiling array-CGH (Roche NimbleGen Inc., Madison, WI, USA). The custom-designed oligonucleotide arrays with 15 bp median probe spacing cover a genomic sequence of approximately 4.7 Mbp on chromosome 7p11.2, including the *EGFR* gene itself, and two reference regions on chromosome 2 and chromosome 10 of about 200 kbp length each [11]. In general, these reference regions show only minor genomic aberrations. A correlation plot comparing the signal intensities between the hybridization from the amplified DNA obtained from one single cell compared to genomic DNA obtained from 500 cells indicates the reliability of the preparation, pre-amplification steps and array hybridization (figure 1c).

Data analyses of cell lines carrying *EGFR* amplifications (MDA-MB-468, A431, and BT20) revealed an amplicon on chromosome 7 that consists of a main part containing the entire *EGFR* gene. This central element is extended in the telomeric direction with a sharp border at the end (figure 1 a, b), which indicated that the amplicon is initiated at a distinct DNA sequence. From single cell analysis of MDA-MB-468 cells displaying different *EGFR* copy numbers, the precise amplicon borders were calculated by the smoothing function of the Quantsmooth algorithm [12]. A further extension of the amplicon was found in MDA-MB-468 cells with the highest copy number gain (32 copies), showing an additional sequence extension on the centromeric site (figure 1b). The data were validated by quantitative SYBR-green PCR using LINE1 repetitive sequences as an intrinsic constant copy control [13] (Table S3 and S4). This approach allows reproducible absolute DNA quantification in a single cell, which has not been reported so far.

### Description of the EGFR amplicon in single cells by qPCR

**Figure 2:**
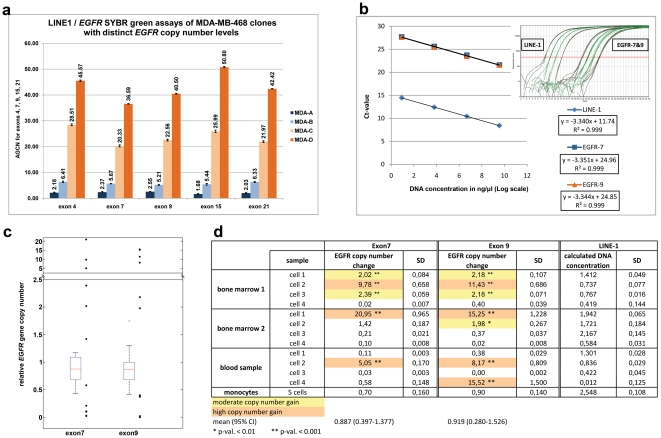
Quantitative PCR. **a:** Quantitative PCR assays for different *EGFR* exons in MDA-MB-468 clones **b:** Calibration curves of the qPCR assays for the LINE-1 control and *EGFR* exons 7 and 9, which indicate that amplification efficiencies of the control as well as sample DNA are similar. **c, d:** Results of the qPCR analysis of exon 7 and 9 in single tumor cells from cancer patients. **c:** Boxplots of qPCR measurements of 10 non-tumourous cells from 3 different patients. The median of the values measured in non-tumour cells is about 0.9 (horizontal line in boxes). Values above 95% confidence limits 1.377 and 1.679 are considered to be gain of exon 7 and 9, respectively. The single black dots represent the values measured in tumor cells from patient samples, according to the table given in **d**.

Our next goal was the development of a reliable assay that can assist therapy decisions in clinical routine. The results of the fine-tiling array-CGH have shown that the amplified regions differ in length dependent on the level of amplification of the whole region (figure 1), but in all cases, the *EGFR* gene itself is included. Based on the copy number levels determined by fine-tiling array analysis, we standardized absolute qPCR measurements for the *EGFR* exons 4, 7, 9, 15, and 21 and the non-coding 5′-sequence of the amplicon at position 54,485,000 in a SYBR green assay to accurately describe *EGFR* amplifications in one single cell (Table S5). The PCR efficiency ranged between 98.07% and 99.02% (average: 98.78%, SD 0.36). All assays showed consistent results as presented in figure 2a. In clinical routine, where a simple and robust assay is desirable, the reduction of these PCR reactions to exon 7 and 9 and at the 5′-end-sequence is sufficient.

### Genomic heterogeneity in blood-borne cancer cells and DTC populations from individual patients

To investigate, whether this integral approach developed and optimized by using MDA-MB-468 cells, as model is also suitable for the analyses of patient samples, we tested blood samples and bone marrow samples of patients with metastatic breast cancer. Cells were prepared as previously described [5], [14]. Cytokeratin and EpCAM antibodies are used as markers for the detection of blood-borne or disseminated cancer cells [5]. We isolated several single cells, stained with the antibody A45B/B3, from different patient samples by micromanipulation and performed WGA as described above. The WGA yielded in 1.96–2.84 µg DNA (average: 2.36 µg, SD 0.25), which is comparable with the yield obtained from single cultured cells. WGA was controlled by the use of LINE1 DNA [13], which yielded in a mean of 330 ng (range 3 ng–621 ng, SD 211) amplification product and allowed the precise calculation of the PCR-amplifiable DNA in each sample. From every patient sample, we analyzed four cells by qPCR for exons 7 and 9 (figure 2) and the 5′-end-sequence (data not shown). The values of the *EGFR* measurements in non-tumor cells have a median of about 0.9 in both qPCR assays, which is expected to reflect the normal copy number of 2 (box plots of figure 2c). Compared to these values, one third of the cells showed a high *EGFR* amplification of at least 5-fold (p<0.0001), and about 25% of the samples showed a gain between 2- and 3-fold (p<0.001). The detailed results for the single cells are shown in figure 2c and 2d. One sample did not show consistence between the measurements in exons 7 and 9. For this sample, we performed additional qPCR measurements on exons 4, 15, and 21, which all show a normal copy number for the respective locus. No single tumor cell presented with a “normal” gene copy number. Cancer cells without amplification of the EGFR gene showed low copy number values (<0.7). These values are rather expected to be caused by variation in the WGA procedure than by technical artifacts of the qPCR, since the calculated starting amount of DNA from most samples applied to the qPCR reaction (>40 pg) is sufficient for reliable measurements and is in the range of the calibration curves as shown for LINE1 intrinsic control (figure 2d).

## Discussion

The aim of the study was the development of a protocol that allows the quantitative genomic analysis of single tumor cells. Primary tumor cells of epithelial origin are heterogeneous. It has been speculated that only a small proportion of these cells show specific “stem cell – like” features and may give rise to metastases (reviewed in [15]). These cells may be characterized by specific genomic aberrations that would provide them with growth advantages and a more aggressive phenotype. This view is supported by data from EGFR inhibitor studies as such, EGFR-mutated adenocarcinoma represent a unique clinical entity necessitating molecular diagnostics for therapy guidance [1]. Moreover, the generation of therapy resistant cell clones harboring additional EGFR mutations or amplifications during therapy as well as the overexpression and amplification of EGFR regulating genes, e.g. ERFI1, have already been reported [2], [16] Therefore, the genomic investigation of single tumor cells that either reside in the blood or in the bone marrow may help to improve predictive molecular diagnostics. Particular in breast cancer, EGFR signaling seems to be upregulated in basal-like tumors (triple-negatives), especially in cells with stem cell like features [6], [7], [17], [18]. Preliminary experimental data exist that benetits from EGFR targeted therapy by cetuximab or laptinib are related to EGFR amplification level [6].

Several research groups have investigated the possibility to analyze a single tumor cell on the molecular level [19], [20]. It has been shown that the genome wide detection of aberrations by the use of BAC arrays is possible in leucocytes and cell line cells [21]. Furthermore, the studies of Stoecklein et al indicated a divergence between the primary and systemic disease [19] in esophageal cancer patients, which strikingly shows the need of the analysis of single disseminated tumor cells.

In our study, we chose the MDA-MB-468 cell line model to develop the protocol because of different stable EGFR amplification levels in different clones of this cell line. By this, these cells somehow mimic the heterogeneous *in vivo* situation and serve as a well balanced control for the determination of gene copy levels. We can show that the amplified DNA obtained from a single cell is of sufficient quality to be used in a high-resolution fine-tiling array analysis. The profiles of single cells show more background, but compared to profiles from genomic DNA of the cell population the same amplicon borders can be clearly identified. Genome wide arrayCGH analysis gives an excellent overview about the genome wide aberrations within a single cell, but the quantification of the genomic changes is limited. A qPCR approach cannot be used to screen for new aberrations, but enables us to quantify known, potentially clinical relevant amplification and deletions. By the use of the qPCR technique, differences in gene copy number could be clearly distinguished as well on the level of genomic DNA of a pool of cells as on the amplified DNA obtained from one single cell. We could show that cells obtained from an individual patient are heterogeneous concerning their EGFR gene amplification. The analysis of only one or two cells per blood sample can therefore lead to results that may not reflect the true clinical situation. Analysis of as many cells as possible is essential to identify those cells that may not be targeted by a specific therapy due to the heterogeneity of the cell population. This finding, if confirmed in a larger cohort of samples, could provide new insights in the biology of metastasis and may help to explain failure of targeted therapies in a significant proportion of patients.

The use of a quantitative PCR assay displays a robust and cost-effective method that can be established under routine conditions. Compared to the use of fine-tiling-microarrays, which are more cost intensive and less robust concerning the interpretation of the data, this methods could potentially be used in clinical routine for the molecular investigation of single cells. This method for the first time displays a straightforward approach to quantify the DNA amount of a specific genomic region from one single cell.

By this, we present a reliable method for the absolute quantitative determination of gene copies in single cancer cells isolated from peripheral blood or bone marrow of cancer patients. This method may not be limited to investigate the biology of disseminating tumor cells, but may also become a new tool for the assessment of single cell genomics with wide applicability in many areas of experimental research. Moreover, future clinical applications can be envisaged. Beyond the investigation of the *EGFR* gene, our method can be adapted to assess other molecular targets (e.g. HER2 [21]), and to analyze cancer cells in other cytological samples, where the low percentage of tumor cells limits the genomic analyses by current methods.

## Materials and Methods

### Ethics Statement

From patients who provided blood samples, written informed consent has been obtained. From bone marrow samples collected after autopsy, written approval was given by the family members. The use of medical records, blood and bone marrow was approved by the ethics committee of the Medical Board Hamburg (reference number #190504).

### Cell Lines and Culturing

The breast cancer cell lines MDA-MB-468, BT-20, and MCF-7 as well as the carcinoma cell line A431 were obtained from the American Type Culture Collection (ATCC) and cultured in DMEM with 5% heat-inactivated fetal calf serum at 5% CO_2_. Cells were grown to 70% to 80% confluency before harvest and transfer to slides for single cell picking.

### Immunocytochemistry

Cells were incubated for 45 minutes with the primary antibody A45B/B3 (Micromet, Munich, Germany), followed by a washing step and a 30 minutes incubation with a rabbit-anti-mouse bridging antibody (Z0259, Dako, Glostrup, Denmark). Detection has been performed with the monoclonal mouse-APAAP complex (Dako, Glostrup, Denmark) and BCIP/NBT as chromogenic substrate according to the manufacturer's protocol (BioRad, Hercules, CA, USA).

### Patient samples

Patient material was obtained from the University Medical Center Hamburg-Eppendorf, Germany. Archival bone marrow samples on cytospins were used. From patients who provided blood samples, written informed consent has been obtained. These samples were obtained from patients undergoing autopsy and of which the family members had given written approval to take bone marrow samples for research purposes. This procedure has been approved by the local ethical committee. From patients who provided blood samples, written informed consent has been obtained.

### Enrichment procedure for blood-borne cancer cells and DTC

Bone marrow samples and peripheral blood samples have been processed by a Ficoll-density gradient to enrich mononuclear cells and possibly epithelial tumor cells as previously described [12]. Shortly, bone marrow samples were obtained from the upper iliac crest by needle aspiration and stored in heparin-treated tubes. Mononuclear cells including tumor cells were separated by Ficoll-Hypaque density-gradient centrifugation with a density of 1.077 g/ml. 7×10^5^ cells were cytospun onto glass slides, dried at room temperature and stored at −80°C.

### Transfer of single cells

#### 1. Silanisation of glass sticks

The glass sticks with a diameter of 1.9–2.3 mm were rinsed for one hour in 85°C in a solution of 10% Neodisher Laboclean FT (Dr. Weigert, Hamburg, Germany). HPLC-clean water was used for all dilution and washing steps. After drying, the sticks were swayed in H_2_SO_4_ (98%, Merck, Darmstadt, Germany) for one hour at room temperature and rinsed in water. Sticks were allowed to dry for 15 minutes at 110°C and subsequently swayed in 0.5% octadecyltrichlorsilane in octane fraction (Fluka, Erlangen, Germany) for two hours. After incubation in 96% ethanol for one hour, residues of ethanol and silane were removed by rinsing with water thoroughly and vigorously. Sticks can be stored dry and dark for up to eight weeks.

#### 2. Lysis buffer

The lysis buffer is composed of 0.25% N-lauroyl sarcosine, 2 M guanidinium thiocyanate, 0.01 M sodium citrate pH 7.0, and 1% dimethylsulfoxide in HPLC-clean water. The buffer was sterilized by filtration through a 0.2 µm PES filter (VWR, Darmstadt, Germany) and stored at −20°C until use.

#### 3. Stick preparation

Before use, DTT was added to the lysis buffer at a final concentration of 60 mM. The complete lysis buffer was diluted 1∶10 with HPLC-clean water. To one end of each glass stick, 0.2 µl of complete lysis buffer was applied and allowed to dry at room temperature until complete dryness. The remaining salts now form the so-called ‘Lysospot’.

#### 4. Single cell picking

Subsequently, cells were collected by the use of a micromanipulator: The microinjector Celltram Vario and micromanipulator TransferMan NKII (Eppendorf Instruments, Hamburg, Germany) were equipped with a custom-designed, flexible capillary customTip type III with an inner diameter of 40 µm and a bevelled end (45°), developed in close collaboration with Eppendorf Instruments. Cells were transferred from the glass slide under visual control by a nuclear fluorescent DAPI-staining to ensure that whole nuclei were captured. To prevent loss of material during transfer and cell lysis, cells were placed directly out of the capillary on the silane-coated glass stick carrying the Lysospot. Due to the silane coating, DNA binding to the glass will be prevented. Furthermore, the salts of the Lysospot are concentrated to a very small area because of the changes in surface tension due to the coating. The complete Lysospot becomes activated by resolution due to the aqueous surrounding during the transfer of the manipulated cell. The cell-containing glass stick was transferred into a 200 µl PCR reaction tube containing 9 µl of sample buffer of the Genomiphi V2 amplification kit (GE Healthcare Europe, Freiburg, Germany) and transferred to −80°C for 15 minutes. After thawing, 0.1 µl protease solution (Qiagen, Hilden, Germany) was added, followed by an incubation step of 15 minutes at 50°C for protein digestion and an additional incubation step of 15 minutes at 75°C for enzyme deactivation.

### Whole Genome Amplification

Whole genome amplification was performed with the Genomiphi V2 kit (GE Healthcare Europe, Freiburg, Germany) according to the manufacturer's protocol with the following modifications: The glass stick remains in the reaction tube during amplification. The amplification reaction was allowed to run for 150 minutes at 30°C in a total volume of 20 µl. The WGA product was cleaned up with NucleoSEQ spin columns (Macherey-Nagel, Düren, Germany) and DNA concentrations were measured by the Nanodrop1000 (Peqlab, Erlangen, Germany).

### Microsatellite Detection

The allele status of the polymorphic repeats was investigated by PCR amplification followed by separation on either a 2% agarose gel or capillary electrophoresis. The reactions were performed in a total volume of 10 µl under the conditions given below (Table S1) on a Mastercycler gradient (Eppendorf Instruments, Hamburg, Germany). The annealing temperature was adapted according to the primers used as shown in Table S2. The separation was performed using a four-color laser induced fluorescence capillary electrophoresis system (AbiPrism 3130; Applied Biosystems, Wilmington, GE, USA) utilizing GeneScan Standard ROX-500 for fragment length evaluation. Evaluation was performed using Genemapper v2.03 evaluation software (Applied Biosystems, Wilmington, DE, USA).

### Fine Tiling Array Analysis

The fine tiling array was designed by Nimblegen (Roche NimbleGen Inc., Madison, WI) as described before [22] according to our custom parameters (Table S6). Bases that are part of repetitive elements have been removed from being considered for probe design. Furthermore, the selected probes were not allowed to contain ambiguous nucleotide codes. Additional criteria were: Annealing temperature of 76°C, probe length between 50–75 bp, the probe was allowed to only match once in the whole genome according to Human genome assembly March 2006 (hg18), http://genome.ucsc.edu (Table S6), and the probes ideally started with an offset of 15 bp. With these specifications, about 395.000 probes can be spotted on one array. The borders of the amplicons for the different MDA-MB-468 cell clones were calculated by the smoothing function of the Quantsmooth algorithm [12] and validated afterwards by quantitative real-time PCR. For this purpose, a total amount of 53 SYBR green assays were designed flanking the calculated start- and endpoints sharing the same specifications. The assays were designed to amplify singular genomic sequences of 50–150 bps in length to ensure specific quantification. Chromosome 2 and chromosome 10 were used as stable reference regions according to Naylor *et al.* [11]. If necessary, the calculated start- and endpoints were corrected based upon the array CGH plot and the qPCR results.

### Quantitative RT-PCR

The quantification of the average gene copy number of *EGFR* was performed using specific primers targeting singular sequences of 50–150 bps within different exons of the *EGFR* gene as given in Table S3. The PCR-reactions were performed on a Mastercycler epgradientS Realplex4 under the conditions given in Table S4in a total volume of 15 µl. Each sample was measured in triplicate. A separate calibration curve was generated for each as say in each run using leukocyte DNA from the same patient ranging from 10–0.15 ng per reaction. DNA concentrations were normalized referring to the constant copy number reference LINE1 [13]. Melting analyses were performed after each run to verify singular product amplification.

*EGFR* gene copy numbers have been determined by calculating the ratio between the DNA amount in the *EGFR* region divided by the DNA amount in the reference region. A normal, diploid gene copy number is ideally reflected by 1 (2n/2n), three copies of the *EGFR* gene would be expected around 1.5 (3n/2n). To calculate the cut-off for calling a sample gained, 95% confidence limits of the reference values were used (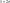). All measurements above the upper limit were considered an *EGFR* gene copy number gain.

## Supporting Information

Table S1 **PCR protocol of the microsatellite PCR.**
(PDF)

Table S2 **Overview of the sequences of the microsatellite primers.**
(PDF)

Table S3 **PCR primer pairs for the qPCR of the** ***EGFR*** **gene.**
(PDF)

Table S4 **PCR protocol for the** ***EGFR*** **-qPCR.**
(PDF)

Table S5 **PCR primer pairs on chromosome 7.**
(PDF)

Table S6 **Contigs for array design.**
(PDF)
