# Supplementary material for: Quantitative High-Resolution Genomic Analysis of Single Cancer Cells
Source: PLoS One. 2011 Nov 30;6(11):e26362. doi: 10.1371/journal.pone.0026362 (PMC3227572; doi:10.1371/journal.pone.0026362)
Supplement: Table S1 — PCR protocol of the microsatellite PCR. (PDF) [file pone.0026362.s001.pdf]

Online table 1 – microsatellite PCR

PCR reaction		PCR programme	
1 µl	10x Gold-PCR-Buffer		
0.8 µl	3.5 mM $MgCl_2$	95°C	10 min.
0.8 µl	8 mM dNTP	35 cycles:	
0.1 µl	100 pmol/µl Primer *forward*	95°C	30 sec.
0.1 µl	100 pmol/µl Primer *reverse*	56 / 62°C	30 sec.
0.1 µl	AmpliTaq-Gold (0.5U)	72°C	30 sec.
0.5 µl	200mM TMAC		
4 µl	DNA (5ng/µl)	72°C	7 min.
ad 10µl	Aqua dest.	4°C	∞
