# Supplementary material for: Quantitative High-Resolution Genomic Analysis of Single Cancer Cells
Source: PLoS One. 2011 Nov 30;6(11):e26362. doi: 10.1371/journal.pone.0026362 (PMC3227572; doi:10.1371/journal.pone.0026362)
Supplement: Table S2 — Overview of the sequences of the microsatellite primers. (PDF) [file pone.0026362.s002.pdf]

Online table 2 – microsatellite primers

Name	Locus	productsize (bps)	Forward primer sequence (5'-3' Orientation)	Reverse primer sequence (5'-3' Orientation)	annealing temperature
D7S522	CHR7-q31.2	121	[6-FAM]GCAGGACATGAGATGACTGA	GTTATGCCACTCCCTCACAC	56°C
D8S258	CHR8-p21.3	229	[6-FAM]AGCTGCCAGGAATCAACTGAGAG	GATGCTCACATAAAGGAGGGAGG	
D16S400	CHR16-q21	179	[6-FAM]GGTTCACAATTGGACAGTAT	GAACCCTCCATGCTGACATT	
NEFL	CHR8-p21.2	109	[6-FAM]CCAATACCTGCAGTAGTGCC	GAGCTGCTTAACACATAGGG	
D13S153	CHR13-q14.2	194	[6-FAM]AGGGTTATGTATAACCGACTCC	GTCTAAGCCCTCGAGTTGTGG	
D17S855	CHR17-q21.31	151	[6-FAM]GGATGGCCTTTTAGAAAGTGG	ACACAGACTTGTCCTACTGCC	
D10S541	CHR10-q23.31	161	[6-FAM]CACCACAGACATCTCACAACC	CCAGTGAATAGTTCAGGGATGG	
D16S402	CHR16-q23.3	112	[6-FAM]GTACCCATGTACCCCCAATA	CAAAGCACCACATAGACTAA	
D16S422	CHR16-q23.3	122	[JOEE]GAGAGGAAGGTGGAAATACA	GTTTAGCAGAATGAGAATAT	
P53CA	CHR17-p13.1	171	[6-FAM]AAGAAATTCCCACTGCCACTC	GCTGTAGAGTGAAGCTCAGGCT	
CASSR1	CHR7-p11.2	121	[6-FAM]GTTTGAAGAATTTGAGCCAACC	TTCTGTCTGCACACTTGGCAC	
CASSR3	CHR7-p11.2	126	[6-FAM]AGGCCCACAGAGGAGATAACAGA	CAGGTGTGGTAGATGCCAAAGAA	62°C
CASSR4	CHR7-p11.2	197	[6-FAM]GCAACTTATCCAAACCCTGACC	AGAGTGGACTAGGAAATGCTAGGAG	
D7S2429	CHR7-q11.21	218	[6-FAM]CAACTGCCCACACACATCTTTC	TGGGAGCTGGGAGTCAAGTG	
D7S494	CHR7-p11.1	116	[6-FAM]AGCTATGACCACCACTGAACTCAAG	TGAGTCTTTGCAAAACATGCCTG	
D7S499	CHR7-p11.2	216	[6-FAM]GCAGGCTCAGTAAGTGGTTGC	CATGAGTGTTCCTGCTGTTTCCT	
D7S2467	CHR7-p12.1	178	[6-FAM]AAGGAGGAATTACTTGGCTGTGC	GTGGTAACGGTCATCTGTGTTCG	
CASSR2	CHR7-p11.2	169	[6-FAM]CTCGAGGTCTCATCCTCTTTCCC	GCAGAGGTGCACAAAGGAGTAATG	
D7S2550	CHR7-p11.2	269	[6-FAM]TTCATTCAGCTCTCCTCGTCTCAC	CTAAGTTCCATTTGTCTCGGTTCCA	
CASSR6	CHR7-p11.2	158	[6-FAM]AGTTCCTGACTGGGAATTCGAT	TTGGCCAAATTACACACCTTTG	
