# Supplementary material for: Quantitative High-Resolution Genomic Analysis of Single Cancer Cells
Source: PLoS One. 2011 Nov 30;6(11):e26362. doi: 10.1371/journal.pone.0026362 (PMC3227572; doi:10.1371/journal.pone.0026362)
Supplement: Table S3 — PCR primer pairs for the qPCR of the EGFR gene. (PDF) [file pone.0026362.s003.pdf]

Online table 3 – EGFR primers

Assay	Exon	productsize (bps)	Forward primer sequence (5'-3' Orientation)	Reverse primer sequence (5'-3' Orientation)	annealing temperature
qEGFR4	4	119	GGTCAAAGGCTAACGTGCAG	TTGAACCCTAATGCACACGAG	
qEGFR7	7	52	CACCACTCACTGAGACCTTGG	GAAATGGAGGCATGGTAGTCC	
qEGFR9	9	107	CACCGTCATCACCTTCCTTTC	TCCTCCATCTCATAGCTGTCG	58°C
qEGFR15	15	107	CCTACGGGTGAGTGGAAAGTG	ACAAACCTCGGCAATTTGTTG	
qEGFR21	21	124	AGCCATAAGTCCTCGACGTG	GAGAAGACCCTGCTGTGAGG	
