# Supplementary material for: Quantitative High-Resolution Genomic Analysis of Single Cancer Cells
Source: PLoS One. 2011 Nov 30;6(11):e26362. doi: 10.1371/journal.pone.0026362 (PMC3227572; doi:10.1371/journal.pone.0026362)
Supplement: Table S4 — PCR protocol for the EGFR -qPCR. (PDF) [file pone.0026362.s004.pdf]

Online table 4 – EGFR PCR

PCR reaction		PCR programme	
		95°C	15 min.
		45 cycles:	
7.5 µl	2x qPCR Master-Mix. No Rox	95°C	15 sec.
0.4 µl	100 pmol/µl Primer *forward*	58°C	30 sec.
0.4 µl	100 pmol/µl Primer *reverse*	68°C	30 sec.
2 µl	DNA (5ng/µl)		
ad 15µl	Aqua dest.		
		95°C	15 sec.
		60-95°C	20 min.
