# Supplementary material for: Quantitative High-Resolution Genomic Analysis of Single Cancer Cells
Source: PLoS One. 2011 Nov 30;6(11):e26362. doi: 10.1371/journal.pone.0026362 (PMC3227572; doi:10.1371/journal.pone.0026362)
Supplement: Table S5 — PCR primer pairs on chromosome 7. (PDF) [file pone.0026362.s005.pdf]

Online table 5 – chromosome 7 primers

Assay	Locus on chrom 7	productsize (bps)	Forward primer sequence (5’-3’ Orientation)	Reverse primer sequence (5’-3’ Orientation)	annealing temperature
WT-F01	54474587	108	CAGTCAGCACATTGACACTGG	AAGTGTGGACCAGCTCTGTCTC	62°C
N-WT-F02	54474908	251	TCACCCAAGTCGTGTATGTCAG	CCACTCTCGAAATTGTTCAGC	60°C
N-WT-F05	54475821	111	TTCTTTGCACATTAAATTGTTTG	AGAGGAATAGGCAGATCAATGG	60°C
N-WT-F03	54476585	50	AAGAAAGGTGATGAGCATGGAC	CCCTTAAATTTGAGAACAAGTGG	60°C
N-WT-F04	54476975	174	GAAAGCATTTCAGCCTTTCAC	CACATGTTCAAATGAATTGATGC	60°C
N-WT-F01	54477208	212	AACCAGCCATATTCCATTCATC	GCCCAAAGTCAGGTCATATACAG	60°C
WT-F02	54477812	109	AGCCAGCTCTTTGATTCTTGG	CAAAGGAAACCTATTGCAAGC	58°C
WT-F03	54478118	209	TCTGTAGCTCATGGATTACTTGG	TACCCGTGGGCTGTGTACC	58°C
0107-F01	54478282	132	CCATGATTCGGGATATGGTC	GGACTCAAACATCTTTAGCCAAC	60°C
N-WT-F06	54478348	257	ACTGTTCTTGGGTAGATTGTTCTG	TGTTGGTCGTTGTATTTGTTTC	60°C
WT-F05	54478766	219	GGCTCCCATGATTCTCCTG	GGGCAACACTGAGCATTAAG	60°C
N-WT-F07	54480737	50	TTTAGAAGATGGATTGGTGAGGAG	CCTGTTCTTGTTCTGACCATTG	60°C
0107-F02	54480749	73	ATTGGTGAGGAGCAGACAATG	TGGTTTGACCTGGATTCACTC	60°C
WT-F04	54480805	105	GAATCCAGGTCAAACCATGTG	TACACAGATGCCTGCTCAAAG	60°C
N-0107-F01	54482382	143	GCACTTTGTTTGACTTTGTAGGG	TTGAACTAAGAAACCAAGGAAGC	60°C
HERVK1	54488749	59	CCAAAGAAGAGACTCTGAGAGGAC	AGTCTTGACATGGCTGTTATTGAG	60°C
HERVK2	54491092	268	GAAATCCAAGCTGTATGTTCAATG	ATTAGAAGTCAGCCTAATGCCATC	60°C
0107-F03	54493228	78	CTGCATCCCAGCTACATCAG	ACACCCTGTAAAGCAATGACC	60°C
0106-F01	54493283	300	CGGGTCATTGCTTTACAGG	TGGGTTCTGAAGGATGGTTG	62°C
0106-F03	54494078	65	ATTTGGGAAGAGCCACGAG	AATCCCAGATTTGGGTAGGG	60°C
0107-F04	54494843	121	GCTCACCAGCACTACCTTCTG	AGAAAGCAGCACCTCACACTC	60°C
0106-F02	54494905	60	ACATCCCTAGCACAGGCATC	CAGAAAGCAGCACCTCACAC	60°C
MDA+F01	54617675	160	TGGTTCATCAACTTGGTAGGG	TTCCGCAATAAGCATGTGAG	60°C
MDA+F03	54619874	166	AAATTGGCAGCAACAGCAC	TTCACTGCCTGAAACTGTGG	60°C
MDA+F02	54622489	58	ACCAGTGTCACCACAAATGC	CTTAGCCTTCCCAGTGTTGC	60°C
MDA+F04	54622738	250	CCTGCCTGACTTACCCACTC	TTATGGAAAGGAGCGTGGAG	62°C
0107-E01	55183276	160	CATGGGTATTGAGGCTCTTTG	ATTGTCCACCAGACAGCTGAG	60°C
MDA+E01	55185453	65	CTGCGTAGGACCTTGCTTTC	GACGTTGTGAGGAGGTAGGC	60°C
0107-E02	55185501	124	TACCTCCTCACAACGTCCATC	TGAAGCCAGAATACCACCAAG	58°C
0107-E03	55187598	115	GTTTGAATGTGGTTTCGTTGG	CCTGTGAGCTGAAGAGTGAGG	58°C
N-MDA+R01	55187603	122	AATGTGGTTTCGTTGGAAGC	GAGCAAAGGTTCCCTGTGAG	60°C
MDA+E02	55187612	245	TCGTTGGAAGCAAATGTGTC	CTTACCAGGCAGTCGCTCTC	62°C
N-MDA+R06	55188053	100	TACCATGCCTCCATTTCCTTAC	GTCCTAATGACGGACATCACAG	60°C
MDA+E03	55193283	284	CGTGTGGGTCAGGTACTTTG	CTCATAACGAGGCTGCTTCC	62°C
N-MDA+R05	55193362	99	CACATCTTATCACAGGGACCAG	GTGAGCACCTGCATGTCTATTC	60°C
MDA+E04	55194763	209	TGCTCTTTGCTTCCATGTTG	CATCTATCCACCACCCAACC	62°C
N-MDA+R03	55194833	124	GTTGTGTCACCACCAGATCAAG	CAACCTGTCCCTCTATCCATTC	60°C
N-MDA+R02	55194935	59	GAATGGATAGAGGGACAGGTTG	ACTTCAAAGGTGTGGGTTCATC	60°C
N-MDA+R04	55195242	139	AAGGTGGTCTGGAGAAACAAAG	GCGTAATCCCAAGGATGTTATG	60°C
N0106-E1	55768337	55	CCTCCTATCCAATGGTCTCTCTC	TTCCTCCTCTGCAAGTAAGTCAG	60°C
0106-E01	55768858	221	CTACGGGCAGAGGGTTCAG	CACAGACAGTTCGTGCAAATC	62°C
N0106-E2	55769143	107	GTCTCAAAGTAAATGTGGGATGTG	TCACCAAACCATATCATATTCAGC	60°C

Assay	Locus on chrom 7	productsize (bps)	Forward primer sequence (5'-3' Orientation)	Reverse primer sequence (5'-3' Orientation)	annealing temperature
0106-E02	55769696	124	GCCACATCAGAGCCATAAGC	TTCCAAGAAGGTCTGTGAGGAC	62°C
N0106-E3	55770062	265	TTTCCCACCTAAAGATAGGATTTG	TATTGAGTCATTGCTGTACCCATC	60°C
N0106-E4	55770747	224	TGGCTGTCTGTGTATGTCTGTATG	AGGGAGAGTATTCACACATGCTTC	60°C
N-WT-E03	55806048	144	TACCAACAGTTCTCCCTCATCC	AGTCTGCGGTGTAGAGGAAATC	60°C
N-WT-E01	55807107	149	GGGACCACATCTTACTCTCCAG	ACAGCACCTTCCTCCTTACATC	60°C
WT-E01	55807123	91	CTCCAGAAGCCCTGACATTG	CTTCCTCCTATCCCTCACTGC	58°C
N-WT-E02	55807185	71	AGGAGAGTGCAGTGAGGGATAG	ACAGCACCTTCCTCCTTACATC	60°C
N-WT-E05	55808624	288	TCCTCAAAGATTGGTTTGTTTG	TCCAATATCCAAGAGGCAGAAC	60°C
N-WT-E06	55808790	91	AGTTCTGCCTCTTGGATATTGG	CACAAATCTGTTCTGACACATGG	60°C
N-WT-E04	55808792	120	ATATAGATTCGGCAGCCTTCAG	TCCAATATCCAAGAGGCAGAAC	60°C
WT-E02	55809200	208	CTGGGAGGACAAGGTAAGAGG	AATGGTGGAGTCACAGCTCAC	62°C
