# Supplementary material for: Quantitative High-Resolution Genomic Analysis of Single Cancer Cells
Source: PLoS One. 2011 Nov 30;6(11):e26362. doi: 10.1371/journal.pone.0026362 (PMC3227572; doi:10.1371/journal.pone.0026362)
Supplement: Table S6 — Contigs for array design. (PDF) [file pone.0026362.s006.pdf]

Online table 6 – Contigs for array design according Human genome assemly March 2006 (hg18), http://genome.ucsc.edu

Chromosome	Assembly accession nr.	Start position	End position	Fragment lenght	Overlap with previous assembly (kb)
2	AC018890	175.272.764	175.446.728	180 kb	
7	AACC02000111	51.551.170	53.166.847	1.16 mb	
	AC073318	53.085.403	53.247.862	162 kb	81
	CH236957	53.179.720	55.702.834	2.52 mb	68
	AC091812	55.692.954	55.815.917	122 kb	10
	AC092647	55.813.918	55.977.808	164 kb	2
	AC092579	55.975.809	56.094.766	119 kb	2
	AC092101	56.092.767	56.131.641	39 kb	2
	AC006970	56.129.667	56.265.748	136 kb	2
10	AC079269	23.123.769	23.319.773	196 kb	
